# Identification and Validation of Key Genes Associated With Systemic Sclerosis-Related Pulmonary Hypertension

**DOI:** 10.3389/fgene.2020.00816

**Published:** 2020-07-24

**Authors:** Ji-Na Zheng, Yang Li, Yue-Mei Yan, Hui Shi, Tian-Tian Zou, Wen-Qi Shao, Qiang Wang

**Affiliations:** ^1^Department of Dermatology, Zhongshan Hospital, Fudan University, Shanghai, China; ^2^Department of Stomatology, Zhongshan Hospital, Fudan University, Shanghai, China; ^3^Department of Cardiology, Shanghai Institute of Cardiovascular Diseases, Zhongshan Hospital, Fudan University, Shanghai, China; ^4^Department of General Surgery, Huashan Hospital, Fudan University, Shanghai, China; ^5^Cancer Metastasis Institute, Fudan University, Shanghai, China; ^6^Department of Laboratory Medicine, Zhongshan Hospital, Fudan University, Shanghai, China

**Keywords:** systemic sclerosis, pulmonary hypertension, non-invasive diagnostic biomarkers, weighted gene co-expression network analysis, type-I interferon, gene expression omnibus

## Abstract

Systemic sclerosis-associated with pulmonary arterial hypertension (SSc-PAH) is still a major cause of SSc related deaths. Early diagnosis and prompt treatment are crucial to reduce the mortality of patients with SSc-PAH. To screen the candidate biomarkers and potential therapeutic targets for SSc-PAH, we analyzed the data set (GSE33463 and GSE19617) for confirming key genes in peripheral blood mononuclear cells from SSc-PAH patients. A total of 105 SSc patients from gene expression omnibus (GEO) were included as discovery cohort (*n* = 69) and duplication cohort (*n* = 36) for screening hub genes by weighted gene co-expression network analysis (WGCNA). Furthermore, an independent validation cohort (*n* = 40), including healthy controls, SSc and SSc-PAH patients, was used for further validation by quantitative real-time polymerase chain reaction. The results showed that four key genes, including *IFIT2*, *IFIT3*, *RSAD2*, and *PARP14*, may serve as potential biomarkers in SSc-PAH. Also, they could be independent risk factors for SSc-PAH. In conclusion, the four key genes can be expected to become the potential therapeutic targets and early biomarkers for accurate therapy and diagnosis of SSc-PAH in the future, which also provides promising insights into the pathogenesis of SSc-PAH at the molecular level.

## Introduction

Systemic sclerosis (SSc) is an intractable autoimmune disease characterized by immune dysfunction, vascular disease, cellular inflammation, and dermal fibrosis and involvement of multiple organs including heart, kidney, and lung with high clinical heterogeneity and mortality than other autoimmune diseases (Cappelli et al., 2013; Denton and Khanna, 2017). Although SSc often involves multiple organs, lung diseases, such as pulmonary arterial hypertension (PAH) and interstitial lung disease (ILD), are considered the main causes of mortality (Varga and Abraham, 2007). Among them, SSc related pulmonary arterial hypertension (SSc-PAH) accounts for nearly 15% of SSc-related deaths (Komócsi et al., 2012). Most patients with SSc-PAH present advanced symptoms and severe hemodynamic derangement at the time of diagnosis (Benza et al., 2012; Sobanski et al., 2016).

Epidemiological investigations have indicated that age (≥47) at the diagnostic time of SSc (Chang et al., 2006), diffusing capacity of the lung for carbon monoxide (DLco) (<60%) and course (>3 years) of SSc are high-risk factors for SSc-PAH (Coghlan et al., 2014). In addition, the phenotyping of SSc-PAH can be explained by a pulmonary arterial vasculopathy (group 1), heart involvement (group 2), chronic lung disease (group 3) as well as by pulmonary veno-occlusive diseases-like lesions (group 1′) (Launay et al., 2017). These previous studies can provide insightful viewpoints concerning the epidemiological characteristics and phenotyping data related to SSc-PAH. Recently, research has revealed that patients with older age at SSc-PAH diagnosis usually portend higher mortality, and early diagnosis and prophylactic therapy of SSc-PAH may have more favorable clinical outcomes (Morrisroe et al., 2017). Thus, early diagnosis and preemptive therapy of SSc-PAH are crucial to reducing the mortality of SSc-PAH patients, especially in younger patients.

The occurrence of SSc-PAH is characterized by progressive remodeling of the small- to medium-sized pulmonary vasculature (Chaisson and Hassoun, 2013). Several researches have mentioned that the activation of the innate immune responses is the underlying mechanism of SSc-PAH, which may precede fibrosis and lead to vascular remodeling, fibrosis, and intraluminal microthrombosis through a variety of mechanisms (Dowson et al., 2017). Peripheral blood mononuclear cells (PBMCs) play a key role in the activation of the innate immune response, which can migrate into the dermis and lung, and lead to increased collagen synthesis in nearby fibroblasts (Yamamoto, 2011). Besides, high-throughput technologies including microarray expression profiles and next-generation sequencing of PBMCs have provided powerful weapons for the diagnosis and prognosis of SSc-PAH patients. Therefore, to make full use of expression profiles and sequencing data of PBMCs from SSc-PAH patients and better understand the pathogenesis of SSc-PAH, we utilized bioinformatics analysis for the exploration of hub genes and key mechanism of SSc-PAH.

In this study, weighted gene co-expression network analysis (WGCNA) was utilized to discover correlation patterns among genes and identify the relationship between co-expressed modules and SSc-PAH. Kyoto Encyclopedia of Genes and Genomes (KEGG) pathway enrichment analysis and Gene Ontology (GO) analysis were also applied to explore potential mechanisms. Specifically, we validated the expression levels and diagnostic accuracy of the four genes including radical S-adenosyl methionine domain containing 2 (*RSAD2*), interferon-induced protein with tetratricopeptide repeats 2 (*IFIT2*), interferon-induced protein with tetratricopeptide repeats 3 (*IFIT3*), and poly (ADP-ribose) polymerase family member 14 (*PARP14*) in an independent validation cohort by quantitative real-time PCR (qRT-PCR) and receiver operating characteristic curve (ROC). Our findings may point to the potential candidate genes for accurate therapy of SSc-PAH and provide powerful evidence for a better understanding of the pathogenesis of SSc-PAH.

## Materials and Methods

### Overall Study Designs

**FIGURE 1:**
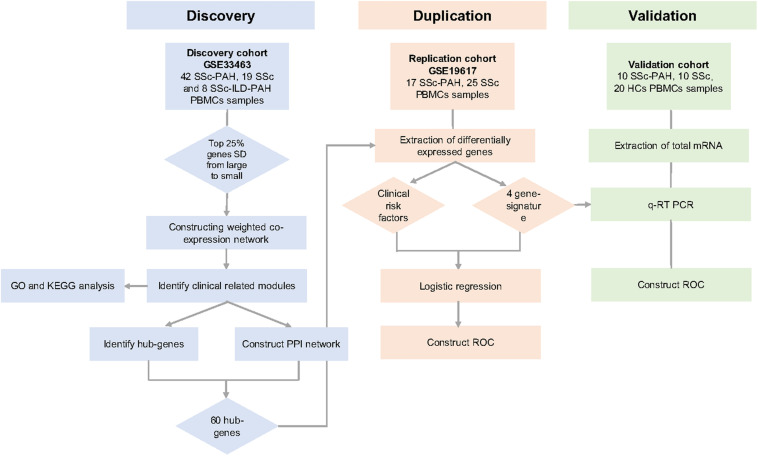
Overall study design. We identified the clinical traits related key modules in GSE33463 by weighted gene co-expression network analysis (WGCNA). Then, we identified 60 hub-genes in the saddlebrown and purple module through the protein-protein interactions (PPI) network. Then, we determined the overlapped four genes between 30 hub-genes and differently expressed genes (DEGs) in GSE19617. Univariate logistic regression (ULR) and multivariate logistic regression (MLR) analysis were utilized to determine independent risk factors for systemic sclerosis-related pulmonary arterial hypertension (SSc-PAH). Through the receiver operating characteristic curve (ROC) analysis, we evaluated the diagnostic capacity of these genes in the GSE19617 and validation cohort. At last, we predicted the potential mechanisms of SSc-PAH by using the Kyoto Encyclopedia of Genes and Genomes (KEGG) pathway and Gene Ontology (GO).

The overall study design was shown (Figure 1). First, highly connected modules and genes were identified in the discovery cohort utilizing the WGCNA approach. Second, the highly relevant co-expression modules were determined by the correlation between the eigengene modules and clinical traits in the discovery dataset. Third, the DEGs were screened between SSc samples and SSc-PAH samples in an independent dataset GSE19617. Next, overlapped genes were determined between DEGs and key module and were validated in an independent validation cohort. Besides, univariate logistic regression (ULR) and multivariate logistic regression (MLR) analysis were utilized to identify which hub genes or clinical factors had a statistically significant effect on SSc-PAH. Finally, ROC was utilized to assess the diagnostic capability of overlapped hub genes in the GSE19617 and validation cohort.

### Patients

A total of 20 patients with a positive diagnosis as SSc according to ACR/EULAR 2013 classification criteria was recruited at Zhongshan Hospital (Fudan University, Shanghai, China) (Van Den Hoogen et al., 2013). Patients presenting pulmonary vascular resistance ≥3 Wood units, mean arterial systolic pressure (mPAP) > 25 mmHg and pulmonary capillary wedge pressure (PCWP) ≤ 15 mm Hg were defined as PAH (*n* = 10) (McLaughlin et al., 2009). Twenty people with no history of pulmonary, autoimmune, cardiovascular, or other diseases were recruited as healthy control subjects (HCs). The study was approved by the Zhongshan Hospital Research Ethics Committee. Written informed consent was acquired from all subjects.

### RNA Extraction and qRT-PCR

A 2–3 ml sample of blood was collected and PBMCs were isolated by human PBMCs separation medium (Axis-Shield, Norway). TRIzol Reagent (Invitrogen, United States) was utilized to extract the total RNA from PBMCs samples. The total RNA was reversely transcribed into cDNA by PrimeScript RT Master Mix (TaKaRa, Japan) according to the manufacturer’s instructions. One microliter of cDNA was used for qRT-PCR with TB Green Premix Ex Taq (TaKaRa, Japan) and specific primer pairs (Table 1). The expression of each gene was normalized to the geometric mean of β-actin to control the variability in expression levels and calculated as 2^–Δ^ ^Δ^ ^*CT*^ [ΔΔCT = (C_*T*_ of gene) − (C_*T*_ of β-actin) − (C_*T*_ of HCs)], in which C_*T*_ represents the threshold cycle for each transcript.

**TABLE 1:** The primers sequence.

Primers	Forward (5′-3′)	Reverse (5′-3′)
*IFIT2*	AAGCACCTCAAAGGGCAAAAC	TCGGCCCATGTGATAGTAGAC
*IFIT3*	TCAGAAGTCTAGTCACTTGGGG	ACACCTTCGCCCTTTCATTTC
*RSAD2*	CAGCGTCAACTATCACTTCACT	AACTCTACTTTGCAGAACCTCAC
*PARP14*	TGTTAGTGGAGAACATAAGTGGC	TGAATGGTGCTTGGTACAATCAT
β*-actin*	CGCGAGAAGATGACCCAGAT	GGGCATACCCCTCGTAGATG

### Gene Expression Profiles of SSc Patients and Processing

Gene expression profiles (GSE33463, GSE19617) were acquired from Gene Expression Omnibus (GEO)^1^ (Edgar et al., 2002). GSE33463 includes 140 PBMCs samples from 42 SSc-PAH patients, 30 idiopathic pulmonary hypertension (IPAH) patients, 19 SSc patients, and 8 SSc patients with complicated by ILD and PAH, compared to PBMCs from 40 healthy individuals. Its platform is Illumina HumanHT-12 V3.0 expression Beadchip. 30 IPAH were excluded when the WGCNA was constructed. As for the GSE19617, it contains 21 PBMCs samples from SSc patients and 15 SSc-PAH samples. Its platform is Agilent-014850 Whole Human Genome Microarray. The raw data were preprocessed by the R packages affy (under the R environment, version 3.6.1) and annotate methods, to make normalized expression profiles with official gene names. The demographic and clinical patient descriptions were summarized (Table 2).

**TABLE 2:** Subject characteristics.

Variable	Discovery cohort		Duplication cohort		Validation cohort	
	SSc-PAH	SSc	SSc-PAH	SSc	SSc-PAH	SSc
**Number**	50	19	15	21	10	10
**Age (mean ± SD years)**	58 ± 10	45 ± 12	65 ± 6	50 ± 11	58 ± 9	50 ± 8
**Gender n (%)**						
Male	11 (22)	7 (24)	3 (20)	3 (14)	2 (20)	2 (20)
Female	39 (78)	12 (76)	12 (80)	18 (86)	8 (80)	8 (80)
**Race n (%)**						
African American	8 (16)	0	NA	NA	0 (0)	0 (0)
Asian	2 (4)	0	NA	NA	10 (100)	10 (100)
Caucasian	40 (80)	19 (100)	NA	NA	0 (0)	0 (0)
Hispanic	0	0	NA	NA	0 (0)	0 (0)
**NYHA functional class n**						
**(% of the PAH subjects)**						
I	2 (4)	NA	NA	NA	0 (0)	NA
II	23 (46)	NA	NA	NA	2 (20)	NA
III	24 (48)	NA	NA	NA	8 (80)	NA
IV	1 (2)	NA	NA	NA	0 (0)	NA
6-MWD (mean ± SD)	1058 ± 346	NA	NA	NA	878 ± 317	NA
mPAP (mean ± SD)	NA	NA	43.6 ± 9.0	NA	45.4 ± 7.4	NA
PCWP (mean ± SD)	NA	NA	11.9 ± 3.6	NA	12.4 ± 3.0	NA
PVR (mean ± SD)	NA	NA	513.8 ± 216.6	NA	NA	NA
FVC (% predicted) (mean ± SD)	NA	NA	80.2 ± 17.3	NA	79.8 ± 19.4	NA
DL_*CO*_ (% predicted) (mean ± SD)	NA	NA	46.1 ± 9.8	NA	47.7 ± 11.6	NA
RAP (mean ± SD)	7.8 ± 4.3	NA	NA	NA	8.4 ± 3.7	NA
CI (mean ± SD)	2.7 ± 0.7	NA	NA	NA	3.0 ± 0.7	NA
PVRI	969 ± 553	NA	NA	NA	998 ± 534	NA

*SSc, systemic sclerosis; PAH, pulmonary arterial hypertension; SD, standard deviation; 6-MWD, 6-Minute Walk Test; mPAP, mean pulmonary pressure; PCWP, pulmonary capillary wedge pressure; PVR, pulmonary vascular resistance; FVC, forced vital capacity; DL_*CO*_, diffusing capacity of the lung for carbon monoxide; RAP, right atrial pressure; CI, cardiac index; PVRI, pulmonary vascular resistance index; NA, not available.*

### Construction of WGCNA

First, ranked by diminishing standard deviation (SD), the top 25% of genes were selected for further analysis. Second, the pickSoftThreshold function was utilized to reckon the soft-thresholding power β for constructing modules. The function provided an appropriate β value ranging from 1 to 30 and the value closest to 0.8 was selected by the result of the scale-free topology fit index and the mean connectivity. Once a suitable β value was determined, co-expressed modules were built by the WGCNA algorithms in R software. Next, the adjacency was converted into a topological overlap matrix for calculating the network connectivity of each gene. Then, genes with similarly expressed patterns were classified as diverse modules with the smallest gene size of 50 based on the topological overlap matrix similarity (Langfelder and Horvath, 2008). Finally, the correlation between the module eigengenes and the clinic traits (PAH or ILD) was measured to identify the correlative modules. Gene significance was determined as the absolute value of the correlation between genes and clinical traits. Quantitative measurement of the module relationship was determined as the connection strength between eigengenes in each module and gene expression profiles.

### Function Enrichment Analysis of Genes in the Key Modules

To further understand potential mechanisms and functions, we performed the GO and KEGG enrichment analysis of the genes in the key modules by using clusterProfiler of R software (Yu et al., 2012). The terms with a *P*-value of <0.05 were considered statistically significant.

### Identification of Hub Genes in Key Modules

Hub genes are one cluster of genes with high connectivity in a module and are considered to be functionally significant. To build the protein-protein interaction (PPI) network, we imported the genes in the key modules into the STRING database^2^ and visualized the top 30 hub-genes in each module by Cytoscape (Smoot et al., 2011; Franceschini et al., 2013).

### Validation of Hub Genes

The “limma” package was applied to identify the DEGs between SSc and SSc-PAH in GSE19617. The cutoff value was log_2_FC > |1|, *P*-value < 0.05. Among them, we selected 386 genes with the most obvious up-regulation and down-regulation (*P* < 0.05), which can better show the difference between sample groups. Next, to overlap DEGs and the genes in key modules, we constructed the Venn diagram by using jvenn^3^ (Bardou et al., 2014).

### Statistical Analysis

Statistical analysis was performed using SPSS 22.0 and GraphPad Prism 6.0. ULR and MLR were applied to determine risk factors for SSc-PAH by SPSS 22.0. ROC was utilized to determine the area under the curve (AUC) of each hub gene to evaluate the abilities for the diagnosis of SSc-PAH by SPSS 22.0. The student’s *t*-test was utilized to calculate statistical differences between different groups by GraphPad Prism 6.0. A *P* value of <0.05 was considered statistically significant.

## Results

### Construction of Weighted Co-expression Network

**FIGURE 2:**
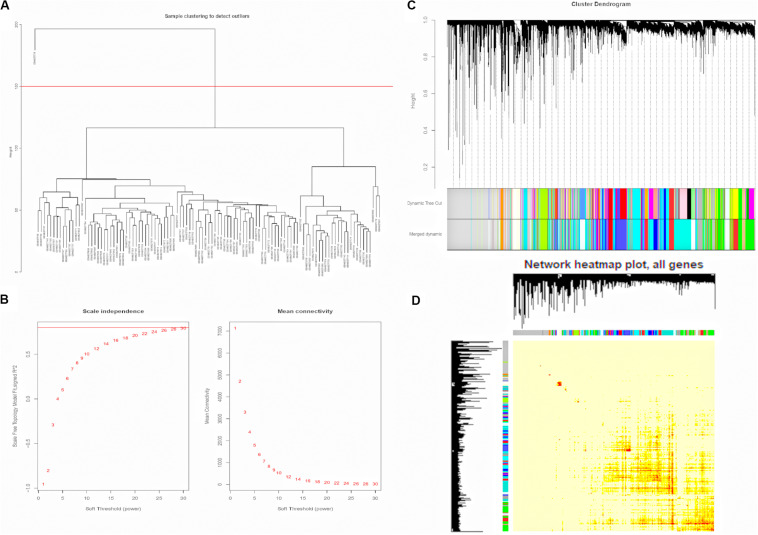
Construction of Weighted Gene Co-expression Network. **(A)** GSM827775 was excluded from the samples. **(B)** Analysis of the scale-free topology model fit index for soft threshold powers (β) and the mean connectivity for soft threshold powers. **(C)** The cluster dendrogram of genes in GSE33463. Each branch means one gene. Each color means one co-expressed module. **(D)** Interactive relationship analysis of co-expression genes. The light color indicates topological overlap, while the darker color indicates a high topological overlap. PAH, pulmonary arterial hypertension; ILD, interstitial lung disease.

To construct the weighted co-expression networks, the top 12200 genes ranked diminishing SD were chosen for WGCNA. Then, cluster analysis was performed (Supplementary Figure S1). One obvious outlier (GSM827775) was removed from the sample cohort, and 109 samples were divided into two clusters for further analysis (Figure 2A). Choosing an appropriate soft threshold power is a crucial step in constructing a WGCNA. A power value of 28, as the closest value of the scale-free topological fit index of 0.8, was identified to construct a dendrogram including 12200 genes (Figure 2B). Then, we merged similar modules to generate 18 modules (Figure 2C). Among them, the maximum module was constitutive of 1094 genes (Turquoise), while the minimum module was constitutive of 55 genes (Violet). Besides, the genes in the gray module represented the genes that were not be included in any module. Next, by plotting the eigengene adjacency heatmap, we revealed the interaction relationships of the 18 modules, indicating that it was highly independent between the co-expressed modules and gene expression in these modules (Figure 2D).

### WGCNA Identifies Key Modules Correlating With SSc-PAH

**FIGURE 3:**
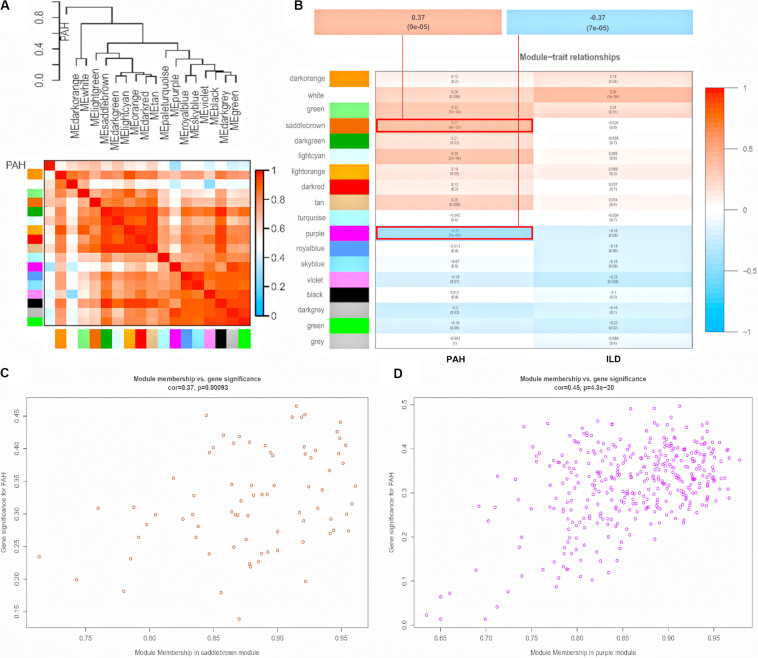
Module-trait relationships. **(A)** Dendrogram and unsupervised hierarchical clustering heatmap of module eigengenes and PAH. **(B)** Heatmap of the correlation between module eigengenes and clinical traits. *P-*value is shown in each color cell coded by the correlation between modules and traits (red indicates positive correlation; blue indicates negative correlation). Scatter plot of module eigengenes in saddlebrown module **(C)** and purple module **(D)**. PAH, pulmonary arterial hypertension; ILD, interstitial lung disease.

To explore the similarity between co-expressed modules, we calculated the association between modules and clinical traits associated with pulmonary phenotypes (PAH and ILD), and clustered eigengenes based on their correlation. As a result, 18 modules were primarily classified into two clusters. PAH and ILD clustered together, indicating similarity between PAH and ILD. The heatmap plotted according to adjacencies showed similar results (Figure 3A and Supplementary Figure S2). Moreover, we measured the association between the modules and clinical traits with *P* value (Figure 3B). We founded that although some modules were correlated both with PAH and ILD, some modules were only correlated with one trait, indicating that they are independent processes. The saddlebrown module and purple module showed a high correlation with PAH in comparison to other modules. The saddlebrown module was the most positive correlation with PAH, while the purple module was the most negative correlation with PAH, suggesting that the saddlebrown module might play a key role in the progression of SSc-PAH, while the purple module might act as suppressor genes. In addition, we analyzed the correlation between module membership and gene significance in the saddlebrown module and purple module, respectively, (Figures 3C,D). The results showed that module membership in the saddlebrown module (*r* = 0.37, *p* = 0.00093) and purple module (*r* = 0.45, *p* = 4.3e-20) was significantly correlated with gene significance for SSc-PAH.

### Functional Enrichment Analysis

**FIGURE 4:**
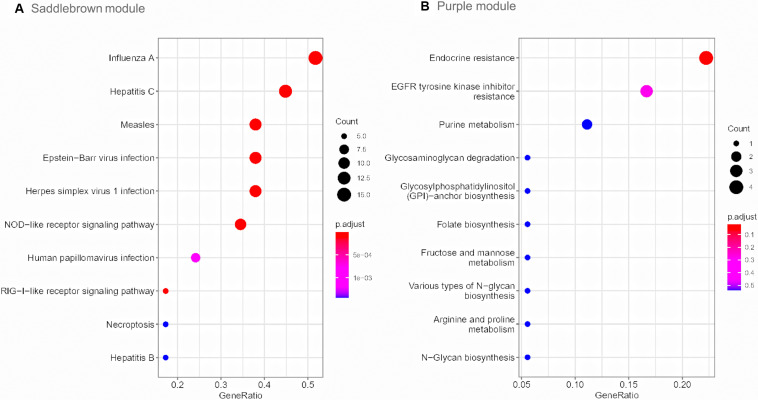
KEGG pathway enrichment analysis. The size of each circle means the amounts of genes. The different color of each circle means the *P*-value. RichFactor means the number of genes in the key module that belong to this pathway divided by the number of genes in the background gene cluster that belong to this pathway. The top 10 KEGG enrichment pathways of genes in saddlebrown **(A)** and purple module **(B)**. KEGG, Kyoto Encyclopedia of Genes and Genomes.

Gene Ontology enrichment analysis revealed that genes in saddlebrown module were mainly distributed in GO:0051607 (defense response to virus), GO:0009615 (response to virus) and GO:0060337 (type I interferon signaling), and which were positively correlated with PAH. Genes in the purple module were mainly enriched in GO:0005622 (intracellular), GO:0043170 (macromolecule metabolic process) and GO:0005488 (binding), which played an important role in metabolic functions (Table 3). Moreover, to understand the biological functions associated with SSc-PAH, we performed KEGG analysis of the genes in the two modules. The saddlebrown module was were mainly distributed in genes encoding for influenza A, hepatitis C and measles pathways (Figure 4A). On the other hand, genes in the purple module mostly encompassed genes with endocrine resistance, EGFR tyrosine kinase inhibitor resistance, and purine metabolism (Figure 4B). These modules demonstrated a positive correlation with PAH, suggesting that the levels of these pathways increase as SSc- PAH advances. In summary, we identified 18 co-expressed modules in PBMCs from SSc-PAH patients by WGCNA. Among them, Type I interferon related genes correlated closely with SSc-PAH while macromolecule metabolic genes showed a strong association with ILD.

**TABLE 3:** GO enrichment analysis of genes in co-expression modules.

Term ID	Term ontology	Count	*P*-value	Term name
**Saddlebrown module**				
GO:0051607	BP	29	8.55E-43	Defense response to virus
GO:0009615	BP	31	1.89E-42	Response to virus
GO:0060337	BP	20	3.09E-34	Type I interferon signaling pathway
GO:0071357	BP	20	3.09E-34	Cellular response to type I interferon
GO:0034340	BP	20	1.05E-33	Response to type I interferon
GO:0045071	BP	11	1.55E-18	Negative regulation of viral genome replication
GO:0043900	BP	18	2.81E-18	Regulation of multi-organism process
GO:0034341	BP	15	3.71E-18	Response to interferon-gamma
GO:0048525	BP	12	1.29E-17	Negative regulation of viral process
GO:1903901	BP	11	1.29E-16	Negative regulation of viral life cycle
**Purple module**				
GO:0005622	CC	217	2.60E-59	Intracellular
GO:0043170	BP	181	2.91E-59	Macromolecule metabolic process
GO:0005488	MF	215	6.00E-57	Binding
GO:0044424	CC	212	6.00E-57	Intracellular part
GO:0071704	BP	190	1.11E-56	Organic substance metabolic process
GO:0019222	BP	152	1.11E-56	Regulation of metabolic process
GO:0044260	BP	172	1.52E-56	Cellular macromolecule metabolic process
GO:0044464	CC	224	8.41E-56	Cell part
GO:0005623	CC	224	1.04E-55	Cell
GO:0008152	BP	191	2.62E-55	Metabolic process

*GO, gene ontology; BP, biological process; CC, cellular component; MF, molecular function.*

### Identification and Validation of Hub Genes in the Key Modules

**FIGURE 5:**
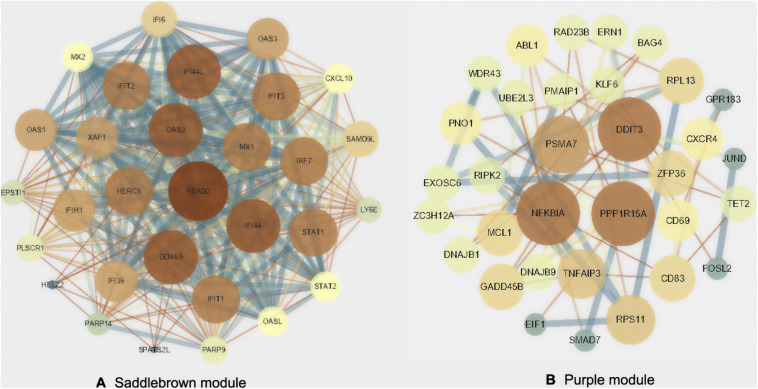
Protein-protein interactions network. Visualization of the network connections among the most connected top 30 genes in the saddlebrown module **(A)** and purple module **(B)**.

**FIGURE 6:**
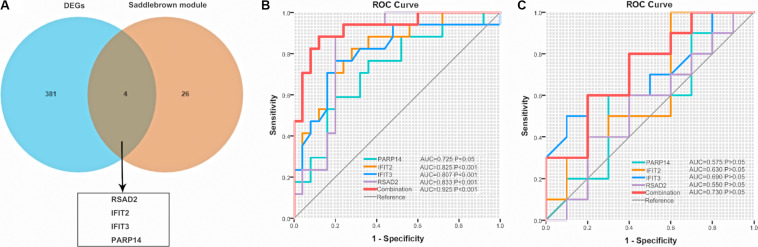
Venn diagram and ROC. **(A)** Common genes between DEGs in GSE19617 and the saddlebrown module. **(B)** ROC of the four genes for the diagnosis of SSc and SSc-PAH in the GSE19617 or the validation cohort **(C)**, respectively. ROC, receiver operating characteristic; SSc, systemic sclerosis; PAH, pulmonary arterial hypertension; DEGs, differently expressed genes; AUC, area under the curve.

**FIGURE 7:**
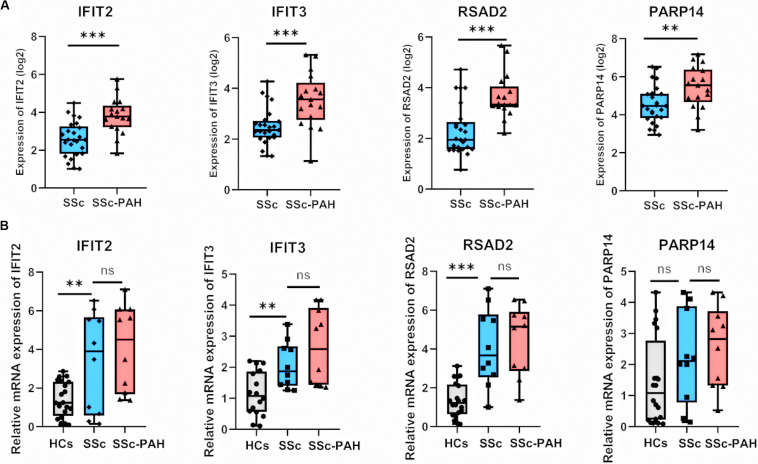
Validation of hub genes in duplication cohort and validation cohort. **(A)** Relative expression of IFIT2, IFIT3, RSAD2, and PARP14 in 25 SSc and 17 SSc-PAH patients for the datasets of GSE1961. Boxplots indicating the median, log 2 (gene expression value) and 25–75% percentiles. **(B)** Validation of IFIT2, IFIT3, RSAD2, and PARP14 in the validation cohort (20 healthy controls, 10 SSc patients and 10 SSc-PAH patients). The results were similar to the above. ***P < 0.001, **P < 0.01, * *P* < 0.05. (Student *t*-test). SSc, systemic sclerosis; PAH, pulmonary arterial hypertension; HCs, healthy controls; mRNA, messenger RNA; WGCNA, weighted gene co-expression network analysis.

We visualized the top 30 genes ranked by node degree in saddlebrown module and the purple module for further analysis, respectively (Figure 5). Also, we used another dataset GSE19617 to testify the expression levels of the 30 hub genes in the saddlebrown module, because it is positively correlated with PAH and better for further validation and detection. We defined the cutoff as logFC > | 1| and *P* < 0.05 to obtain DEGs. We overlapped the DEGs and genes in the saddlebrown module by Venn diagram. As a result, four overlapped hub genes (*IFIT2*, *IFIT3*, *RSAD2*, and *PARP14*) were shown in DEGs and saddlebrown modules, indicating that the four genes we are looking for are highly related to the SSc-PAH (Figure 6A). We also revealed the expression levels of four hub genes in SSc and SSc-PAH PBMCs samples of GSE19617, which indicated the expression level of *IFIT2*, *IFIT3*, *RSAD2*, and *PARP14* was significantly higher in PBMCs from SSc-PAH patients than that from SSc patients (Figure 7A). Besides, we included 10 SSc patients, 10 SSc-PAH patients and 20 healthy controls from Zhongshan hospital (Fudan University, Shanghai, China) as an independent validation cohort to verify the expression of the four hub genes. The mRNA levels of *IFIT2*, *IFIT3*, *RSAD2*, and *PARP14* were significantly higher in PBMCs from SSc patients than those from HCs (Figure 7B). Although it was not statistically significant in the difference of relative mRNA expression in PBMCs between SSc patients and SSc-PAH patients due to limited samples, there was a trend that the mRNA expression levels of *IFIT2*, *IFIT3*, *RSAD2*, and *PARP14* in PBMCs from SSc-PAH patients were higher than that from SSc patients.

### Four Hub Genes Could Be Good Diagnostic Biomarkers for SSc-PAH

To further explore the relationship between these four hub genes (*IFIT2, IFIT3, RSAD2*, and *PAPR14*) and clinical risk factors (age, gender, ILD, forced vital capacity (FVC)% predicted, DL_*CO*_% predicted) in patients with SSc-PAH, we applied the ULR analysis in duplication cohort. We found that four genes, age, and DLco (% predicted) could become independent risk factors for SSc-PAH (Table 4). Next, to explore the diagnostic capabilities of these four genes for patients with SSc-PAH, we performed ROC in the duplication cohort. The results revealed that the AUC was 0.802 for *IFIT2*, 0.807 for *IFIT3*, 0.833 for *RSAD2*, 0.725 for *PARP14*, and 0.925 (*P* < 0.001) for the combination of these four genes (Figure 6B). Then, we validated the potential utility of the four-gene signature as new diagnostic biomarkers of SSc-PAH in our independent validation cohort. The AUCs for *IFIT2*, *IFIT3*, *RSAD2*, *PARP14*, and combination of them when distinguishing SSc-PAH patients from HCs were 0.630, 0.690, 0.550, 0.575, and 0.720 (Figure 6C). In summary, the above results indicated a moderate diagnostic accuracy of four genes as a new biomarker for SSc-PAH. Next, we used MLR analyses to explore the correlation between significant SSc-PAH risk factors and the four genes. However, the results of MLR analyses indicated that four genes and risk factors were not significantly correlated with the diagnosis of SSc-PAH (Table 4).

**TABLE 4:** Univariate and multivariate logistic regression of hub genes and clinical traits.

Hub genes/clinical traits	Univariate logistic regression			Multivariate logistic regression		
	OR	95% CI	*P*-value	OR	95% CI	*P*-value
Gender	1.538	0.268, 8.818	0.629			
Age	1.196	1.069, 1.337	0.002***	0.620	0.103, 3.717	0.601
ILD	1.636	0.384, 6.968	0.505			
FVC (%predict)	0.970	0.932, 1.009	0.134			
DLCO (%predict)	0.890	0.820, 0.966	0.005**	0.979	0.173, 5.864	0.980
PAPR14	2.222	1.124, 4.394	0.022*	0.978	0.172, 5.543	0.997
RSAD2	3.705	1.492, 9.200	0.005**			
IFIT2	4.157	1.161, 10.734	0.003***	1.108	0.890, 1.378	0.363
IFIT3	3.705	1.492, 9.200	0.005**	1.048	0.959, 1.146	0.298

CI, confidence interval; OR, odds ratio. ***P < 0.005, **P < 0.01, *P < 0.05.

## Discussion

Pulmonary arterial hypertension is a devastating condition characterized by proliferative remodeling of the small pulmonary arteries, which causes significant disability and often results in premature death (Humbert et al., 2019). Although the quality of life for PAH patients have been greatly improved by advanced medical means, annual mortality remains high at ∼10% in IPAH. Prognosis is even worse in certain subgroups such as SSc-PAH patients (Humbert et al., 2010a, b). Therefore, to better prevent and treat the SSc-PAH patients, it is necessary to develop novel biomarkers and potential targets at the molecular level. In the present study, the key genes for SSc-PAH patients were screened using the data set (GSE33463 and GSE19617). The independent validation cohort, including HCs, SSc and SSc-PAH patients, were used for further validation. Our research demonstrated that the four key genes including *IFIT2*, *IFIT3*, *RSAD2*, and *PARP14* were identified and validated as the potential biomarkers for SSc-PAH. The results provided novel insights into the pathogenesis of SSc-PAH. Besides, the four genes could be the therapeutic targets for accurate therapy of SSc-PAH in the future.

By deeply and systemically reanalyzing the GSE33463, we identified the saddlebrown module and purple module that were most correlative with SSc-PAH. Further analysis of the saddlebrown module indicated potential mechanisms for SSc-PAH and confirmed key genes in PBMCs from SSc-PAH. Based on KEGG and GO analyses, the saddlebrown module was mainly involved in type-I interferon signaling and response to virus, which are consistent with previous findings (Vaughan et al., 2000; Hamamdzic et al., 2001; Namboodiri et al., 2004; Pandey, 2004; Farina et al., 2014; Farina et al., 2017; Efthymiou et al., 2019). Amongst pathogens, human cytomegalovirus (HCMV) is considered as triggers of SSc, which causes elevated anti-HCMV antibodies in serum (Vaughan et al., 2000; Namboodiri et al., 2004; Pandey, 2004; Efthymiou et al., 2019). HCMV also causes vasculopathy similar to the pathogenesis of SSc-PAH. For instance, Hamamdzic et al. demonstrated patients infected by CMV might present the formation of neointima characteristics by autoimmune vasculopathy (Hamamdzic et al., 2001). Also, SSc patients might show severe symptoms of virus infection mainly related to Epstein-bar virus (Farina et al., 2014; Farina et al., 2017). For the type-I interferon signaling pathway, some researches have revealed that the type-I interferon signaling pathway-related genes, such as STAT4 and IRF5, are associated with a significant risk of SSc occurrence (Dieude et al., 2009; Gourh et al., 2009; Ito et al., 2009; Rueda et al., 2009). Moreover, several studies measuring the type-I interferon signature in PBMCs from SSc patients have consistently revealed that the majority of SSc patients have evidence that type-I interferon related genes were increased (Tan et al., 2006; York et al., 2007; Duan et al., 2008; Higgs et al., 2012; Guo et al., 2015; Bodewes et al., 2018). Furthermore, several case reports have emphasized a possible application of type-I interferon therapy to PAH (Anderson et al., 2014; Fok et al., 2016; Demerouti et al., 2019). Our study has also confirmed the notion that type-I interferon may potentially induce PAH in at least some SSc patients reported by Christmann et al. (2011). Therefore, genes in the saddlebrown module could be well representative of the molecular changes in PBMCs from SSc-PAH patients.

Also, we determined the overlapped four genes between 30 hub-genes and DEGs in GSE19617. Interestingly, through ROC and ULR analysis of four genes, we found that the combination of *IFIT2*, *IFIT3*, *RSAD2*, and *PARP14* may potentially act as a novel biomarker for SSc-PAH. Further analyses of these genes in the validation cohort confirmed that they could diagnose SSc-PAH patients. The expression levels of *IFIT2*, *IFIT3*, *RSAD2*, and *PARP14* tend that relative mRNA expression was higher in SSc-PAH patients than that in SSc patients. We also found that the upregulation of these genes could be independent risk factors for SSc-PAH. *RSAD2* is considered an interferon-stimulated gene that takes part in innate immunity and plays an important role in antiviral responses (Jang et al., 2018). Several studies have demonstrated that *RSAD2* showed hypomethylation and overexpression in blood from SSc patients (Tan et al., 2006; Bos et al., 2009; Assassi et al., 2010; Bodewes et al., 2018). Consistent with a more recent study that investigated the contributions of DNA methylation to the pathogenesis of SSc, a subset of 27 genes with concomitant differential expression was detected in whole blood from 27 twin pairs discordant for SSc, including *RSAD2* (Ramos et al., 2019). Both *IFIT2* and *IFIT3* are the members of the *IFIT* family and are induced by type I interferon (Daffis et al., 2010). Recent evidences have demonstrated that *IFIT2* and *IFIT3* may contribute to the pathogenesis of autoimmune diseases, such as systemic lupus erythematosus, sjögren’s syndrome and pemphigus (Sezin et al., 2017; Bodewes et al., 2018). *PARP14*, also called collaborator of STAT6, is a member of *PARP* superfamily, which was described to associate with the transcription factor STAT6 (Goenka and Boothby, 2006). A previous study has demonstrated that *PARP*-1 is crucial for PAH development (Meloche et al., 2014). Thus, further studies that focus on the potential function of *PARP14* in patients with SSc or SSc-PAH are needed.

Compared with the previous studies, our study provides new insights into the pathogenesis of SSc-PAH. Lenna et al. (2013) analyzed microarray expression profiling from HC PBMCs stimulated with thapsigargin and validated that activating transcription factor-4 and -6 (ATF4 and ATF6), immunoglobulin-heavy-chain binding protein (BiP), and a spliced form of X-box binding protein (XBP1) were elevated in PBMCs from patients with limited cutaneous systemic sclerosis (lcSSc), with the higher levels in SSc-PAH patients. Consistent with our findings, they also revealed that the expression levels of interferon -related genes were significantly increased in PBMCs from lcSSc patients. Similarly, Christmann et al. (2011) analyzed GSE19617 and revealed that C-C motif chemokine receptor 1 (CCR1) and Janus kinase 2 (JAK2) were elevated in patients with lcSSc-PAH compared with HC. The expression level of *MRC1* was elevated exclusively in patients with lcSSc-PAH. Moreover, (Risbano et al., 2010) used GSE22356 to screen DEGs as biomarkers for SSc-PAH and confirmed that interleukin-7 receptor and chemokine receptor 7 as DEGs in patients with SSc-PAH. However, the genes identified by Risbano et al. (2010) came from a microarray cohort of SSc or SSc-PAH patients with smaller sample size (*n* = 20), which raiseed the possibility of overfitting their classifier. However, our study identified the key genes in SSc-PAH in three independent cohorts, and determined the key genes as potential biomarkers by different methods.

A limitation of this study is that despite the value of validation cohort for reliability, our limited samples from largely middle-aged female patients of Chinese ancestry cannot represent the general population. Therefore, our findings may not generalize total SSc or SSc-PAH patients. Further studies with large sample size are needed for the validation, justification, and generalization of our results. Moreover, lack of the phenotyping data of SSc-PAH is a major limitation in this study. Due to the different phenotypes of SSc-PAH, pathological the mechanisms of SSc-PAH are highly heterogeneous. However, datasets included SSc-PAH patients’ samples are very few in the public databases, not to mention detailed phenotyping data of SSc-PAH patients. Therefore, it can be challenging to determine the dominant mechanism in a particular patient due to the limitation of phenotyping data. Despite these limitations, these findings highlight candidate genes and underlying mechanisms for further research.

## Conclusion

In conclusion, we identified the key genes (*IFIT2*, *IFIT3*, *RSAD2*, and *PARP14*) associated with SSc-PAH. The upregulation of these genes may be the independent risk factors for SSc-PAH. Our current study provides reliable evidence that these genes are associated with the occurrence of SSc-PAH. Bioinformatics analysis revealed these key genes were involved in response to virus, cellular response to type I interferon and type I interferon signaling pathway. In the future, there remains a strong need for well-designed clinical studies with more exhaustive strategies, more homogenized populations, and larger samples size to study the underlying function of these genes in the development of SSc-PAH.

## Data Availability Statement

Publicly available datasets were analyzed in this study. Discovery cohort and duplication cohort (GSE33463 and GSE19617) were downloaded from GEO (http://www.ncbi.nlm.nih.gov/geo/).

## Ethics Statement

The studies involving human participants were reviewed and approved by Zhongshan Hospital Research Ethics Committee. The patients/participants provided their written informed consent to participate in this study. Written informed consent was obtained from the individual(s) for the publication of any potentially identifiable images or data included in this article.

## Author Contributions

J-NZ, YL, and QW designed the study. W-QS collected the blood samples. J-NZ, YL, and T-TZ did the statistical analyses. Y-MY and HS prepared the figures. J-NZ, YL, Y-MY, HS, W-QS, and QW reviewed the results and wrote the manuscript. All authors have made an intellectual contribution to the manuscript. All authors read and approved the final manuscript.

## Conflict of Interest

The authors declare that the research was conducted in the absence of any commercial or financial relationships that could be construed as a potential conflict of interest.

## Footnotes

1 https://www.ncbi.nlm.nih.gov/geo/

2 http://string-db.org

3 http://jvenn.toulouse.inra.fr/app/example.html

## Supplementary Material

FIGURE S1 Hierarchical clustering dendrogram of the samples. The clinical traits were shown at the bottom.

FIGURE S2 Dendrogram and unsupervised hierarchical clustering heatmap of module eigengenes and ILD. ILD, interstitial lung disease.
