# Supplementary figures and images for: Identification and Validation of Key Genes Associated With Systemic Sclerosis-Related Pulmonary Hypertension
Source: Front Genet. 2020 Jul 24;11:816. doi: 10.3389/fgene.2020.00816 (PMC7393672; doi:10.3389/fgene.2020.00816)

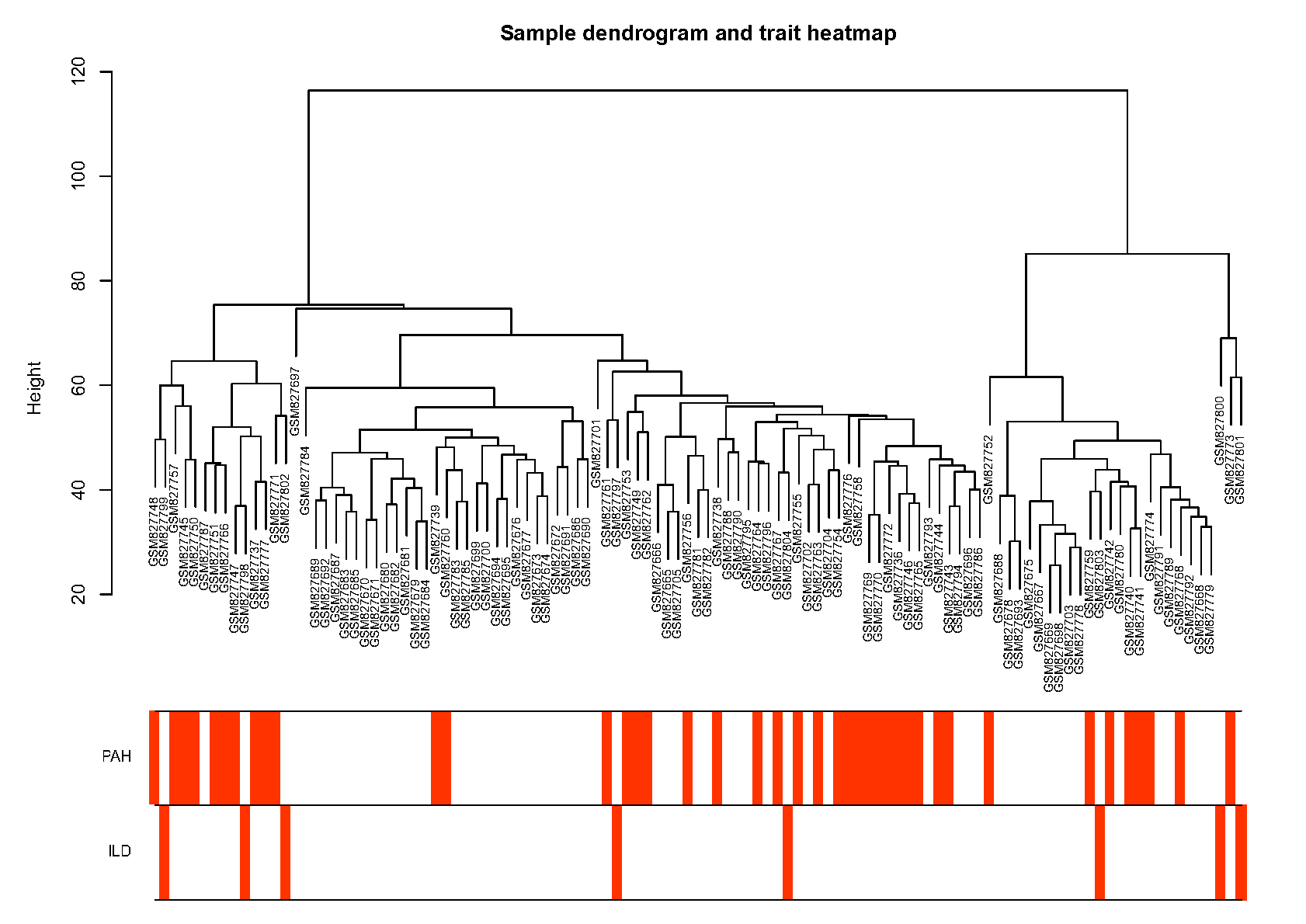

Supplement: FIGURE S1 — Hierarchical clustering dendrogram of the samples. The clinical traits were shown at the bottom. [file Image_1.TIF]
